# Chiral recognition of ephedrine: Hydrophilic polymers bearing β-cyclodextrin moieties as chiral sensitive host molecules

**DOI:** 10.3762/bjoc.7.177

**Published:** 2011-11-10

**Authors:** Sabrina Gingter, Helmut Ritter

**Affiliations:** 1 Institute of Organic Chemistry and Macromolecular Chemistry II, Heinrich-Heine-University, Universitätsstraße 1, 40255 Düsseldorf (Germany)

**Keywords:** chiral recognition, cyclodextrins, ephedrine, host–guest interaction, stimuli-responsive polymer

## Abstract

In this work we demonstrate chiral recognition of (+)- and (−)-ephedrine using a cyclodextrin-containing polymer. The supramolecular structures obtained by complexation of ephedrine and cyclodextrin were verified by 2-D ROESY NMR measurements. Dynamic light scattering showed clear differences in the mean coil size.

## Findings

**Scheme 1:**
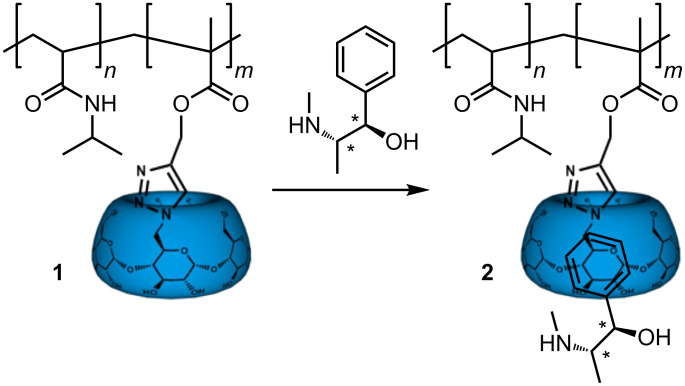
Synthetic pathway to the desired β-cyclodextrin-comprising copolymer and its supramolecular system.

Chiral recognition is an important topic in medical and pharmaceutical applications. The sheer number of publications dealing with chiral and molecular recognition systems underlines the importance of finding reliable recognition systems [1–5]. Particularly, the use of cyclodextrins as chiral host molecules has been the focus of attention for many investigations [6–7]. β-Cyclodextrins (β-CDs) are widely used as selectors for the resolution of enantiomers of chiral drugs in both separation techniques and spectroscopic methods [8–14]. Cyclodextrins are cyclic oligosaccharides comprising six, seven or eight α-1–4-linked D-glucopyranose units. They are used in pharmaceutical, medical and industrial applications, allowing even hydrophobic molecules to become water-soluble [15–16]. However, there are only a few works published dealing with enantiomeric recognition in polymeric systems comprising CD moieties [17–18]. Recently we reported the chiral recognition of synthetic polymers bearing enantiomeric amino acids phenylalanine and tryptophan through complexation with β-CD [6,19]. In this work we are interested in the chiral recognition of the pharmaceutically active (+)- and (−)-ephedrine. Ephedrine is an alkaloid that functions as a decongestant, stimulant and appetite suppressant. Ephedrine is an aromatic amine and belongs to the group of amphetamines. We chose ephedrine as a model compound for the present investigation, as it exhibits chirality and aromaticity and is pharmaceutically relevant (Scheme 1).

Recently we described the monomer synthesis of mono-(6-azido-6-desoxy)-β-cyclodextrin with propargylmethacrylate and the copolymerization with *N*-isopropylacrylamide (NIPAAM) [20]. The properties of the polymer **1** were characterized further by use of size-exclusion chromatography (SEC), dynamic light scattering (DLS) and turbidity measurements.

**Figure 1:**
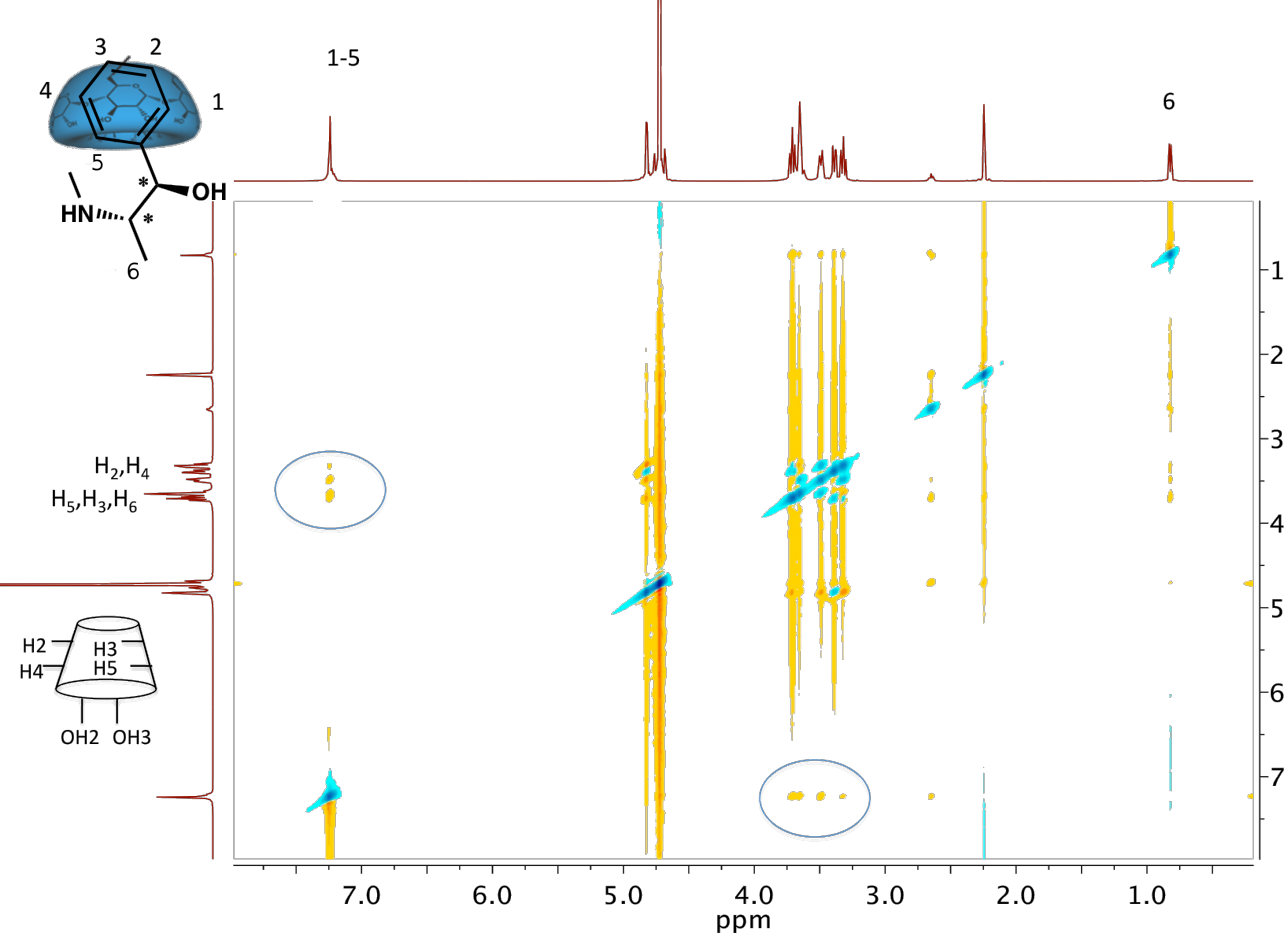
2-D ROESY NMR spectrum of complexed β-CD with (+)-ephedrine.

To confirm the formation of the proposed host–guest structure of β*-*cyclodextrin and ephedrine in principle, 2-D ROESY NMR spectroscopy was performed at room temperature with β-CD in excess. Thus the correlation between the protons of the β-CD moiety and the protons of the (+)-ephedrine aromatic ring was demonstrated (Figure 1). As the correlation of the protons only occurs between the aromatic and the CD moiety, the complex stoichiometry can be considered as 1:1, as it was also found previously [21–22].

**Figure 2:**
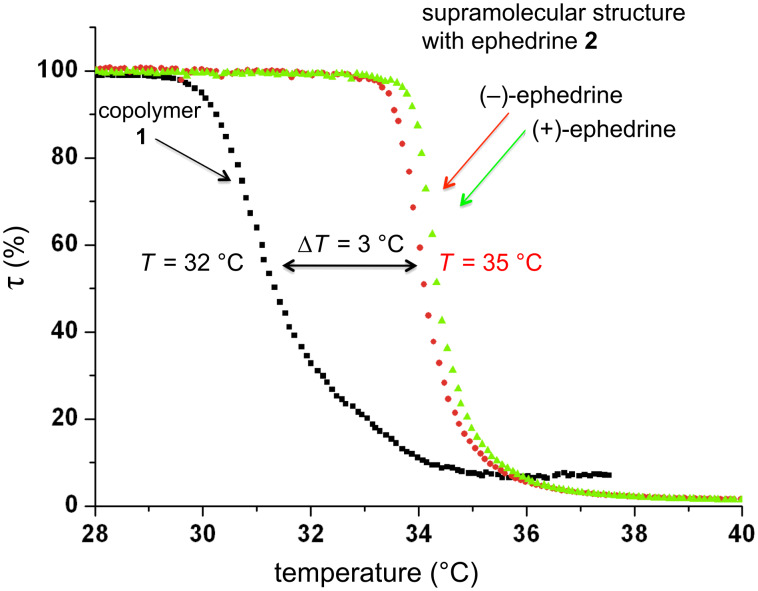
Turbidity measurement showing different cloud points for the CD-comprising copolymer **1** (32 °C) and the supramolecular structure **2** (35 °C).

As reported recently, the CD containing NIPAAm copolymer **1** exhibits lower critical solution temperature (LCST) behavior in water [23]. As expected, the supramolecular system **2** turned out to be soluble in cold water only below the LCST of 32 °C. However, due to the presence of relatively hydrophilic ephedrine in the host–guest system, the LCST rises to 35 °C for polymer **2**, which is 3 °C higher than the value for pure poly(NIPAAM). The increase of LCST takes place because the hydrophobic phenyl ring of ephedrine is incorporated into the CD cavity, and the hydrophilic OH and amino groups are located in the outer water phase. Figure 2 shows the results of cloud-point measurements performed on a turbidity photometer. However, the turbidity measurements only showed the inclusion of ephedrine, and no significant enantiomeric effect was observed.

**Figure 3:**
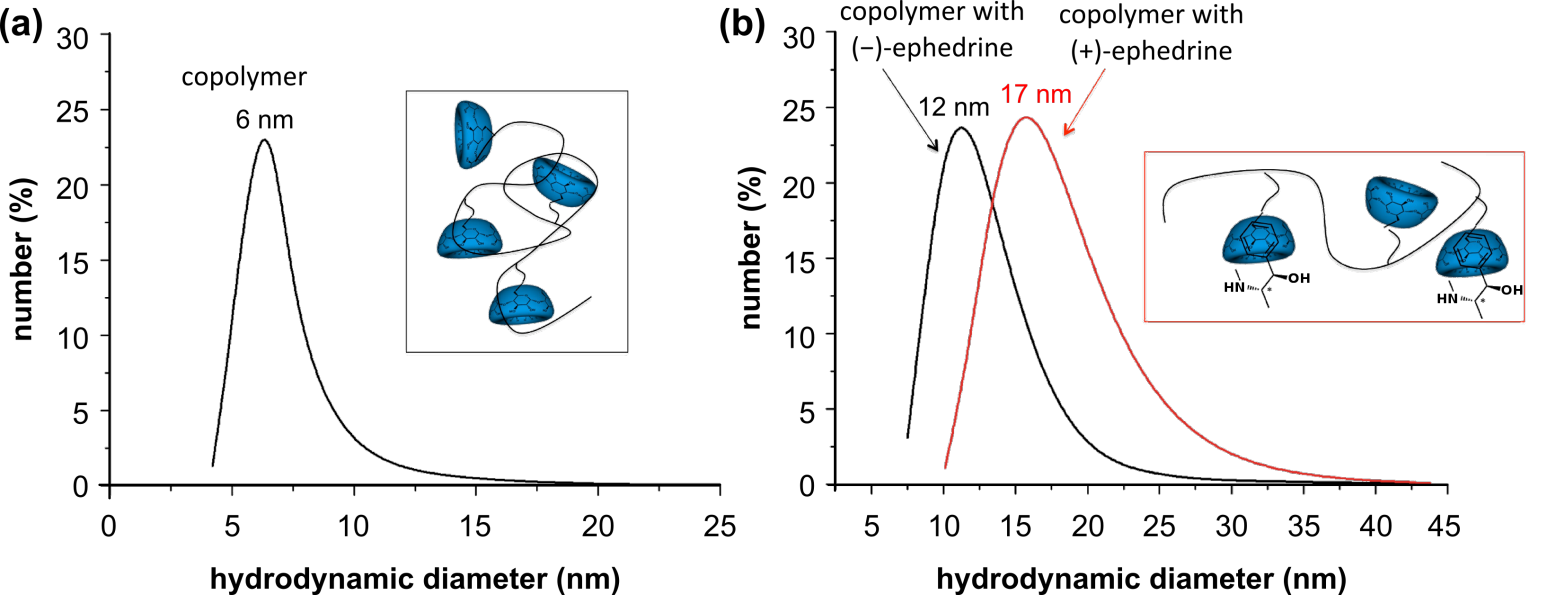
DLS measurement showing hydrodynamic diameters for (a) the CD-comprising polymer and (b) for supramolecular structures with (+)-and (−)-ephedrine respectively.

However, the supposed enantiomeric differences due to diastereomeric effects were expected to influence at least the hydrodynamic diameters of the polymer. Therefore, dynamic light scattering (DLS) measurements of the aqueous solutions were conducted. Indeed, the mean hydrodynamic diameter of polymer **1** extended from 6 nm to 12 nm after complexation of (−)-ephedrine and to 17 nm after complexation with (+)-ephedrine (Figure 3). The reproducible difference of 5 nm in the hydrodynamic diameter of the complexes can be attributed to the different complexation behavior of the two ephedrine enantiomers [24–25].

In conclusion, we showed a characteristic chiral recognition with a CD-containing polymer complexed with (+)- or (−)-ephedrine. The supramolecular structures obtained by complexation of ephedrine and CD were detected by 2-D ROESY NMR measurements. Dynamic light scattering exhibited clear differences of 5 nm in the mean coil size, between the two enantiomers. Therefore we conclude that (+)-ephedrine is preferred in complexation with CD, due to a thermodynamic effect. Additionally turbidity measurements showed a slight difference between the pure copolymer and the supramolecular structure of 3 °C, but no enantiomeric effect was observed.

## Experimental

(1*S*,2*R*)-(+)-Ephedrine hydrochloride and (1*R*,2*S*)-(−)-ephedrine hydrochloride were obtained from Acros Organics and used as received. β-Cyclodextrin (β-CD) was obtained from Wacker-Chemie GmbH, Burghausen, Germany and used after drying overnight in an oil-pump vacuum over P_2_O_5_. ^1^H NMR and ROESY experiments were performed on a Bruker Avance DRX 200 or Bruker AVIII-300 spectrometer operating at 200.13 MHz or 300 MHz with deuteriumoxide 99.9% as solvent. The chemical shifts (δ) are given in ppm with the solvent peak as an internal standard. The ROESY experiments were carried out at room temperature with 30 mg of copolymer **1** and 60 mg of β-CD in alkaline D_2_O. Turbidity experiments were performed on a Tepper cloud-point photometer TP1. The relative transmission of a laser beam with a wavelength of 670 nm was recorded for each experiment. The measurements were performed within a temperature range between 5 and 40 °C and at a heating rate of 1 K·min^−1^, and by using Hellma Suprasil precision cells 110 Q-S and aqueous solutions of copolymer **1** with 5 mg/mL and 2 mg/mL of ephedrine. Critical solution temperatures derived from these experiments were determined at 50% relative transmission. Dynamic light scattering (DLS) experiments were carried out with a Malvern High Performance Particle Sizer HPPS-ET at a temperature of 20 °C in aqueous solutions of copolymer **1** (1 mg/mL) and ephedrine (1 mg/mL). The particle size distribution is derived from a deconvolution of the measured intensity autocorrelation function of the sample by General Purpose Method (non-negative least squares) algorithm included in the DTS software. Each experiment was performed at least five times.
